# VHL Ser65 mutations enhance HIF2α signaling and promote epithelial-mesenchymal transition of renal cancer cells

**DOI:** 10.1186/s13578-022-00790-x

**Published:** 2022-05-03

**Authors:** Xueyou Ma, Zenglai Tan, Qin Zhang, Kaifang Ma, Jun Xiao, Xiong Wang, Yanan Wang, Mengjie Zhong, Yu Wang, Jing Li, Xing Zeng, Wei Guan, Shaogang Wang, Kan Gong, Gong-Hong Wei, Zhihua Wang

**Affiliations:** 1 Department of Urology, Tongji Hospital, Tongji Medical College, Huazhong University of Science and Technology, Wuhan, 430030 China; 2 Department of Urology, the First Affiliated Hospital, Zhejiang University School of Medicine, Hangzhou, 310003 China; 3 Faculty of Biochemistry and Molecular Medicine, Biocenter Oulu, University of Oulu, 90014 Oulu, Finland; 4 Department of Urology, Peking University First Hospital, Beijing, 100034 China; 5 Department of Laboratory Medicine, Tongji Hospital, Tongji Medical College, Huazhong University of Science and Technology, Wuhan, 430030 China; 6 Ministry of Education Key Laboratory of Metabolism and Molecular Medicine & Department of Biochemistry and Molecular Biology, School of Basic Medical Sciences, and Fudan University Shanghai Cancer Center, Shanghai Medical College of Fudan University, Shanghai, 200032 China; 7 Department of Bioinformatics, Center for Translational Medicine, Naval Military Medical University, Shanghai, 200433 China

**Keywords:** VHL mutation, Renal cell carcinoma, VHL c.193 T > C or c.194C > G, HIF signaling, Transcriptional reprograming

## Abstract

**Background:**

Von Hippel-Lindau (VHL) disease is an autosomal dominant genetic neoplastic disorder caused by germline mutation or deletion of the *VHL* gene, characterized by the tendency to develop multisystem benign or malignant tumors. The mechanism of *VHL* mutants in pathogenicity is poorly understand.

**Results:**

Here we identified heterozygous missense mutations c.193T > C and c.194C > G in *VHL* in several patients from two Chinese families. These mutations are predicted to cause Serine (c.193T > C) to Proline and Tryptophan (c.194C > G) substitution at residue 65 of VHL protein (p.Ser65Pro and Ser65Trp). Ser65 residue, located within the β-domain and nearby the interaction sites with hypoxia-inducing factor α (HIFα), is highly conserved among different species. We observed gain of functions in VHL mutations, thereby stabilizing HIF2α protein and reprograming HIF2α genome-wide target gene transcriptional programs. Further analysis of independent cohorts of patients with renal carcinoma revealed specific HIF2α gene expression signatures in the context of VHL Ser65Pro or Ser65Trp mutation, showing high correlations with hypoxia and epithelial-mesenchymal transition signaling activities and strong associations with poor prognosis.

**Conclusions:**

Together, our findings highlight the crucial role of pVHL-HIF dysregulation in VHL disease and strengthen the clinical relevance and significance of the missense mutations of Ser65 residue in pVHL in the familial VHL disease.

**Supplementary Information:**

The online version contains supplementary material available at 10.1186/s13578-022-00790-x.

## Background

The von Hippel-Lindau (VHL) disease is a hereditary, autosomal dominant, neoplastic disease that affects approximately 1/36000–45,500 live births [1, 2]. VHL disease encompasses various types of tumors, including retinal or central nervous system hemangioblastoma (RH or CNSH), clear cell renal cell carcinoma (RCC), pheochromocytoma (PHE), multiple pancreatic cysts or tumors (PCT), epididymal or broad ligament cystadenomas and endolymphatic sac tumor. The leading causes of death are RCC and CNSH [3]. Clinical diagnosis of VHL disease often relies on the presence of classical manifestations and family history. However, in present familial cases, up to 50% of patients have only one manifestation of the disease [4]. Moreover, approximately 20% of patients result from a de novo mutation and do not have a family history [5, 6], which makes genetic diagnosis an irreplaceable supplement to clinical diagnostic criteria, particularly for the early diagnosis of VHL disease. Early identification affected individuals with asymptomatic VHL disease-associated tumors and mutation carriers via genetic recognition and rational medical interventions would improve patients’ survival rates and quality of life. Thus, confirming “Pathogenic” probability of the responsible mutation is particularly essential.

Germline heterozygous mutation of the *VHL* tumor suppressor gene, located on 3p25.3, encoding VHL tumor suppressor protein (pVHL), has been identified as the leading cause of VHL disease. Mutations leading to *VHL* loss cause a number of diseases with divergent features, including VHL disease, sporadic tumors (all tumors associated with VHL disease), familial erythrocytosis type 2, and breast cancer [7, 8]. pVHL is best known as the substrate-binding subunit of an E3 ubiquitin ligase, which binds the transcription elongation factors C and B (elongin C/B) forms the VCB complex, then interacts with Cullin-2 (CUL2) and the RING finger protein RBX1 forming the VCB-CR complex(3). VCB-CR complex targets hypoxia-inducible factor (HIF1α or HIF2α, also known as EPAS1) for polyubiquitylation and subsequent proteolytic degradation in normal physiological conditions [9]. pVHL consists of α-domain and β-domain. The β-domain comprises several β-sheets and binds HIFα via residues 65–117 [10, 11]. *VHL* germ-line mutations leading pVHL dysfunction and accumulation of HIFα will activate the hypoxic gene response and enhance expression of genes involved in ﻿angiogenesis (VEGF), erythropoiesis (erythropoietin, EPO) and ﻿glycolysis and glucose transport (GLUT1) [12, 13]. HIF1α and HIF2α play crucial roles in VHL disease, particularly with respect to ccRCC and hemangioblastoma. Increasing evidence showed that HIF2α is the key driver of RCC progression [14]. Since the *VHL* gene was identified by positional cloning in 1993 [15], over 650 different *VHL* gene mutations have been documented in the Human Gene Mutation Database (HGMD) from reporters around the world. However, the functional study of these *VHL* mutants in pathogenicity is very limited.

In this study, we present the clinical, histological and genetic discoveries of several Chinese families affected with VHL disease and identify heterozygous missense mutations including c.193T > C (alteration of amino acid of VHL protein, p.Ser65Pro or VHL-S65P) and c.194C > G (alteration of amino acid of VHL protein, p.Ser65Trp or VHL-S65W) in *VHL* gene. Furthermore, we have performed intensive functional and mechanistic study of pathogenicity of these VHL mutants and reveal gain of function for the crucial tumor suppressive VHL protein (pVHL). We show how these VHL mutants stabilize HIF2α, and thereby influence HIF2α-target gene transcriptional program as the major mechanism on signaling pathway activation of hypoxia, glycolysis and epithelial-mesenchymal transition in renal carcinoma cells. We thus provide a representative genomic insight into the value of individual amino acid changes for pVHL structural and functional alterations in disease progression.

## Results

### Phenotype and Histopathologic Analysis

**Fig. 1:**
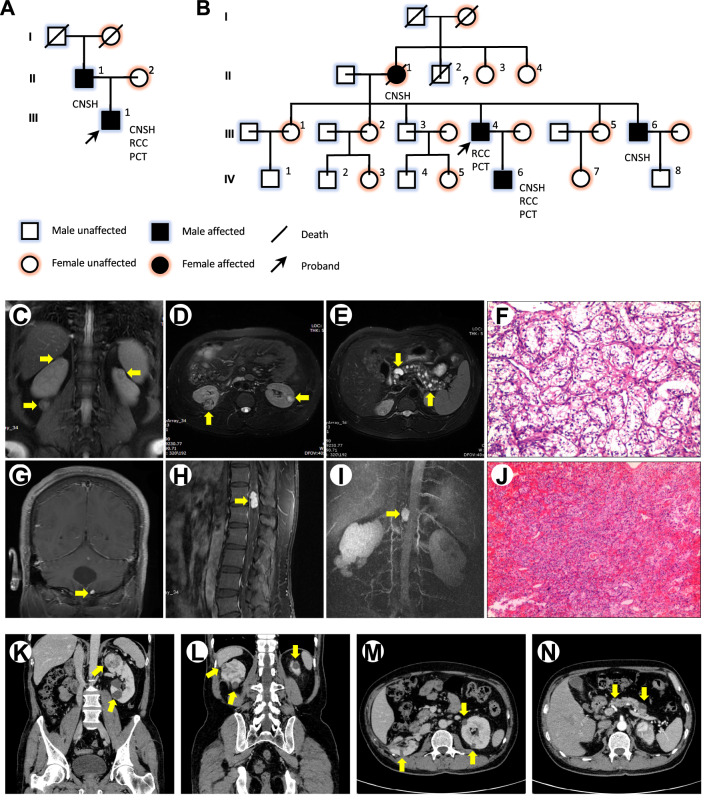
Family pedigree and clinical information. **A** The family pedigree of family 1, c.193T > C (p.Ser65Pro). **B** The family pedigree of family 2, c.194C > G (p.Ser65Trp). An arrow indicates the proband. Squares signify men, and circles women. The filled symbols represent affected individuals. A diagonal line across a symbol indicates a deceased person. CNSH, central nervous system hemangioblastoma; RCC, renal cell carcinoma; PCT, multiple pancreatic cysts or tumors; Pheo, pheochromocytoma. **C**–**J** Imaging and pathological findings of the patient III-1 of family 1. **C**, **D**: abdominal MRI showed multiple occupying lesions in the kidney; **E** multiple pancreatic cysts; F: renal tumor sections from the proband were stained with H&E; **G** hemangioblastomas in the left cerebellar vermis; H,I: Spinal hemangioblastomas at the T12-L1 level; J: Spinal tumor sections from the proband were stained with H&E. **K–N** Imaging of the patient III-4 of family 2. K-M, abdominal CT showed multiple occupying lesions in the kidney; N, multiple pancreatic cysts

The pedigrees and clinical information of the family members are presented in Fig. 1 and Table 1. For the Family 1 c.193 T > C (p.Ser65Pro or VHL-S65P), the proband (Patient III-1, Fig. 1A) was diagnosed as VHL disease at 29 years of age in 2017. The young man has before been diagnosed with hemangioblastoma of the cerebellar vermis and right lateral ventricle (CNSH) at age of 16 years, with epilepsy at age of 23 years and with spinal hemangioblastoma (CNSH) at age of 25 years, with renal cell cancer (RCC) and multiple pancreatic cysts (PCT) at age of 29 years (Table 1). Abdominal magnetic resonance imaging (MRI) of the proband revealed multiple occupying lesions in the kidney (Fig. 1C, D) and multiple cysts in the pancreas (Fig. 1E). MRI of the brain and spine revealed two enhancing lesions in the left cerebellar vermis (Fig. 1G) and at the thoracic and lumbar cord T12-L1 level (Fig. 1H, I), indicating a radiological diagnosis of hemangioblastoma. There was no evidences for diagnosis of RH nor PHE. The renal tumors and spinal T12-L1 tumor were surgically resected. And the postoperative pathological evaluation comfirmed the diagnosis of clear cell renal cell carcinoma (ccRCC) (Fig. 1F) and hemangioblastoma (Fig. 1J).

**Table 1:** Genotypes and phenotypes in 2 Chinese Von Hippel-Lindau disease pedigrees

Patient	Family History	Exon	Nucleotide Change and Consequence	Clinical Type	Age at Diagnosis (y)				
					CNSH	RH	RCC	PCT	PHE
Family 1	+	1	c.193 T > C (p.Ser65Pro)	1					
III-1			Heterozygous		16	–	29	29	–
II-1			Heterozygous		25	–	–	–	–
Family 2	+	1	c.194C > G (p.Ser65Trp)	1					
III-4			Heterozygous		-	–	33	33	–
II-1*			UN		UN	–	–	–	–
III-6			Heterozygous		32	–	–	–	–
IV-6			Heterozygous		22	–	23	23	–

^*^Death before enrollment, age of death is 63y

*CNSH* central nervous system hemangioblastoma, *RH* retinal hemangioblastoma; *RCC* renal cell carcinoma, *PCT* pancreatic cysts or tumors, *PHE* pheochromocytoma, *UN* unknown

The proband’s grandparents (I-1 and I-2, Fig. 1A) were deceased before enrollment. His 57-year-old father (II-1) underwent the operation for cerebellar hemangioblastoma (CNSH) at 25 years of age. The tumors did not relapse. His mother (II-2) was healthy, and her physical examination did not reveal related lesions. Without PHE, the proband and his father were classified as type 1 VHL disease.

For the Family 2 c.194C > G (p.Ser65Trp or VHL-S65W), the proband (Patient III-4, Fig. 1B) was diagnosed as VHL disease at age of 48 years in 2013. He was first diagnosed with multiple RCC and PCT at age of 33 years (Table 1). Abdominal computerized tomography (CT) scan identified multiple occupying lesions in the kidney (Fig. 1K–M) and multiple cysts in the pancreas (Fig. 1N). There was no evidences for diagnosis of CNSH, RH nor PHE. The proband’s grandparents were deceased before enrollment. His mother (II-1) was diagnosed with CNSH. His brother (III-6) was diagnosed with CNSH at age of 32 years. His son (IV-6) was diagnosed with CNSH at age of 22 years, with multiple RCC and PCT at 23 years of age. Without PHE, the Family 2 was classified as type 1 VHL disease.

In comparison, Family-1 (c.193T > C) and Family-2 (c.194C > G) have a certain similarity in phenotype. From the clinic, all patients of these two families presented with Clinical Type 1 disease, including CNSH, RCC, PCT, but not PHE. However, there are several subtle phenotypic differences between S65P and S65W. There are a total of sixteen patients with S65P mutation and seven patients with S65W mutation in our database. The penetrance of RCC was 43.57% (7/16) in S65P, and 57.10% (4/7) in S65W. The average onset age of RCC was 36 years for S65P and 39 years for S65W (Additional file 6: Table S1). However, the reference value of these comparisons is limited due to the relatively small number of cases of rare diseases.

### Detection of *VHL* c.193 T > C and c.194C > G mutations in the families

**Fig. 2:**
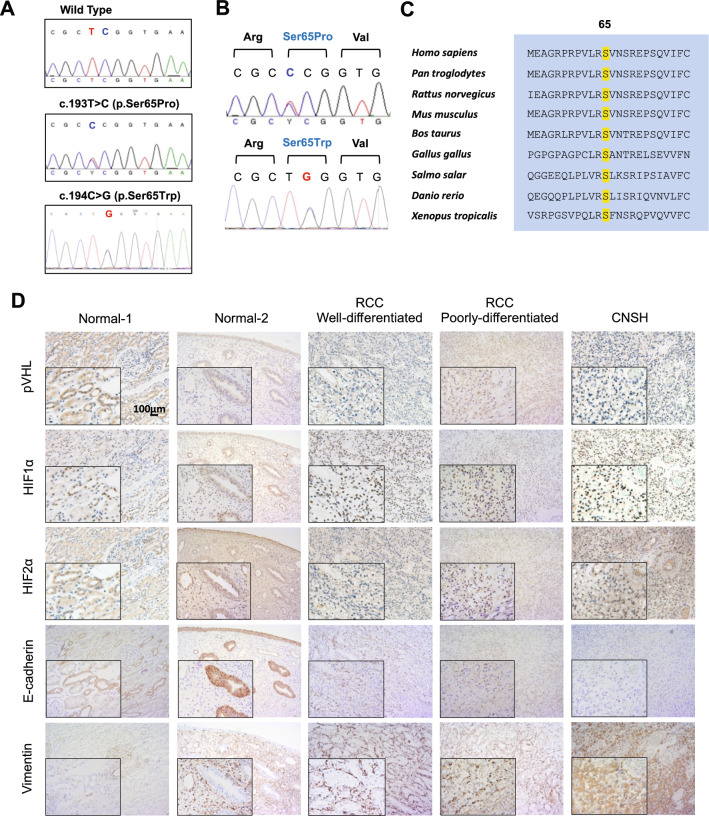
Detection of *VHL* c.193T > C mutation in the family. **A** DNA sequence traces of a representative wild-type allele, a representative heterozygous c.193T > C mutation from the family 1 and c.194C > G mutation from the family2. **B** The mutations were predicted to alter Ser to Pro or Trp at residue 65. **C** Alignment of pVHL sequence from various species indicated the Ser65 residue is highly conserved in VHL proteins across different species. **D** Representative immunostaining results of expression of pVHL, HIF-1α, HIF-2α, Vimentin and E-cadherin in the para-tumor renal tissue (Normal), renal cell carcinoma (RCC, well-differentiated and poorly-differentiated), and hemangioblastoma (CNSH), respectively

DNA sequence analysis revealed that patients who were clinically diagnosed with VHL disease carried heterozygous missense mutation c.193 T > C and c.194C > G in the *VHL* gene for Family 1 and Family 2, respectively, which was not detected in unaffected family members (Table 1; Fig. 2A). No additional insertion, deletion or non-synonymous point mutations of the *VHL* gene were detected.

The nucleotide transversions (c.193T > C) in *VHL* exon 1 was predicted to result in an amino acid alteration at residue 65, replacing Serine with Proline (p.Ser65Pro or S65P) in pVHL, and c.194C > G causing replacement of Serine with Tryptophan (p.Ser65Trp or S65W) (Fig. 2B). Ser65 and its nearby residues are highly conserved among different species (Fig. 2C), suggesting that this residue is important for maintaining the protein’s structure and function. The two mutations were predicted to be “disease causing” by MutationTaster [16] and classified as “pathogenic” variation according to ClinVar [17] of NCBI.

Previous studies demonstrated that pVHL is crucial for the degradation of hypoxia-inducible factor alpha (HIFα) subunits. Moreover, constitutive HIF activation may underlie the angiogenic phenotype of VHL disease-associated tumors, including RCC and hemangioblastoma [18, 19]. We evaluated the expression levels of pVHL, HIF1α and HIF2α in the tissue sections of RCC and hemangioblastoma from the proband (III-1) of Family 1. Immunohistochemical (IHC) staining analysis revealed that both RCC and hemangioblastoma tissues exhibited apparent elevated levels of HIF1α and HIF2α, compared to the para-tumor renal tissue, and also observed that Epithelial-to-mesenchymal transition (EMT) markers Vimentin upregulated and E-Cadherin downregulated in the tumor specimens (Fig. 2D). These data indicate that the VHL-S65P mutation could potentially lead to enhanced HIFα expression, which may play a critical role in VHL disease-associated RCC and hemangioblastoma.

### VHL mutations increase HIF2α protein stability and are sensitive to the ubiquitin proteasome-mediated protein degradation

**Fig. 3:**
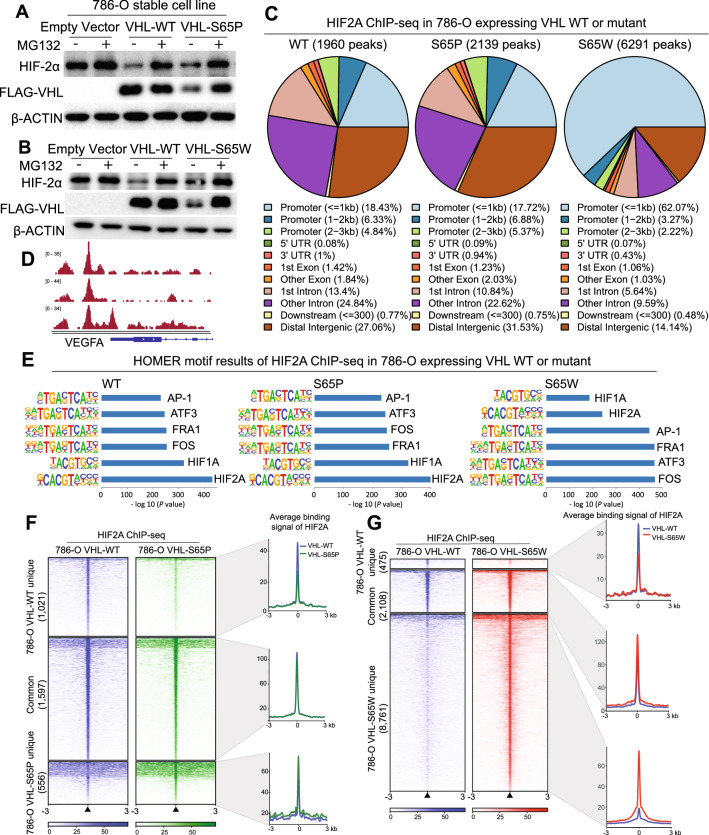
Gain of function of VHL mutations impact genome-wide HIF2α chromatin binding sites in 786-O cells. **A**, **B** Western blot determination of protein expression levels in 786-O cells stably expressing VHL WT and mutants, with or without treatment of the proteasome inhibitor MG132. **C** Genomic annotation of HIF2α ChIP-seq peaks from VHL WT mutant expressing cells. Genomic features of binding peaks were visualized in pie charts, where the demonstrated genomic features, including promoter regions within 1 kb, 1–2 kb and 2–3 kb; gene body (5′UTR, 3′UTR, exons, and introns); downstream elements, and distal intergenic regions. UTR, untranslated region. **D** Visualization of a HIF2α binding site at the promoter of a known hypoxia-regulated gene, VEGFA. **E** The top enriched motifs in the HIF2α ChIP-seq peaks determined by HOMER software. **F**, **G** Heatmap indication of HIF2α chromatin binding intensity based on ChIP-seq reads in 786-O cells expressing VHL WT vs. S65P mutant (**F**) or VHL WT vs. S65W mutant (**G**). Signals within 3 kb around ChIP-seq peak center are demonstrated in a descending order for each clustered HIF2α binding event (common or unique). Plots in the right panels of F or G display average signal of HIF2α binding at the indicated clustered regions

We next sought to explore whether the *VHL* missense mutation VHL-S65P and VHL-S65W show any effects on HIF2α stability. Thus, we generated the stable cell lines, 786-O with ectopic expression of wide type VHL or its mutants, VHL-S65P and VHL-S65W, respectively. Because renal carcinoma is a leading cause of death for patients with VHL disease, we chose to characterize the roles of VHL mutants in the VHL-defective cell model 786-O in subsequent analyses. Western blotting showed protein products of VHL and HIF2α, confirming that the VHL-S65P has a positive impact on HIF2α stability (Fig. 3A and Additional File 1: Fig. S1A). Similarly, the VHL-S65W also indicates a positive effect on stabilizing HIF2α (Fig. 3B and Additional File 1: Fig. S1A). Given that VHL mutations may abolish HIFα binding ability, thereby stabilizing HIFα [20]. To this end, we performed co-immunoprecipitation (Co-IP) assays and found that indeed the interaction between HIF2α and VHL was reduced in the S65P and S65W mutant cells compared with VHL-WT cells, and thus stabilizing HIF2α. (Additional File 1: Fig. S1B). In addition, we used hypoxia mimetics CoCl_2_ and DMOG to treat the 786-O cells. In our experiment, CoCl_2_ treatment induced HIF2α protein level moderately in VHL-WT expressing cells but not in mutant VHL expressing cells, and HIF2α was induced by DMOG treatment in each cell type (Additional File 1: Fig. S1C). To some extent, this is consistent with previous reports showing that CoCl_2_ induces hypoxia through inhibiting the interaction between HIF-α and VHL by direct binding to HIF-α [21] and DMOG directly inhibits PHD leading to HIF-α stability [22].

Of note, either the VHL-S65P or VHL-S65W mutation yields a less stable VHL protein in 786-O, implying that the ubiquitin–proteasome pathway may be involved, which in turn results in VHL protein degradation and thus HIF2α stabilization. To prove this, we added the proteasome inhibitor MG132 in cell culture and found that protein levels of VHL mutants and HIF2α are highly increased as measured by both Western blotting and immunofluorescence assays (Fig. 3A, B and Additional File 1: Fig. S1D). In contrast, MG132 additions show no obvious effects on the protein levels of wild type VHL (Fig. 3A, B and Additional File 1: Fig. S1D), further proving that the VHL mutations are sensitive to the ubiquitin–proteasome system mediated degradation.

### Genome-wide identification of HIF2α target genes and pathways in renal cancer cells expressing VHL variants

To determine how genome-wide chromatin location of HIF2α evolves upon VHL mutation, we next performed ChIP sequencing assays on 786-O cells with ectopic expression of normal or mutated VHL. We identified 2,625 peaks and a less amount (2,174) for HIF2α in VHL-WT or VHL-S65P expressing cells, respectively. In contrast, we identified more peaks (10,889) for HIF2α in the VHL-S65W expressing 786-O cells (Fig. 3C). Comparisons across the data showed that HIF2α binding sites in VHL-S65P cells indicate a slightly increase in the distal intergenic regions while in VHL-S65W cells, HIF2α chromatin occupancies are dramatically enriched in the gene proximal promoter regions (Fig. 3C), suggesting a distinct global shift of HIF2α genomic binding profile. An example of HIF2α ChIP-seq profile at the well-known HIF2α target gene VEGFA [12] is shown in Fig. 3D. Motif analysis of ChIP-seq peaks showed that HIF2α motifs are consistently top enriched in the data from VHL-WT and VHL-S65P cells, respectively (Fig. 3E). In contrast, HIF2α motifs are runner up after FOS/AP-1 motifs in HIF2α peaks derived from VHL-S65W cells (Fig. 3E), demonstrating that the mutations in VHL influence not only HIF2α genome-wide chromatin binding profile but also its transcriptional complex recruitments. We next compared HIF2α binding intensity and found an overall slightly decreased chromatin intensity for HIF2α in VHL-S65P cells (Fig. 3F), and strongly increased intensity in VHL-S65W cells (Fig. 3G). In parallel, we ruled out that this is not due to the amount of ChIP-seq reads, where there are less amount of ChIP-seq reads of 10,209,757 for VHL-S65W HIF2α versus VHL-WT, 14,193,704 or VHL-S65P, 14,998,548 (2 × 150 bp paired-end sequencing; see Methods).

**Fig. 4:**
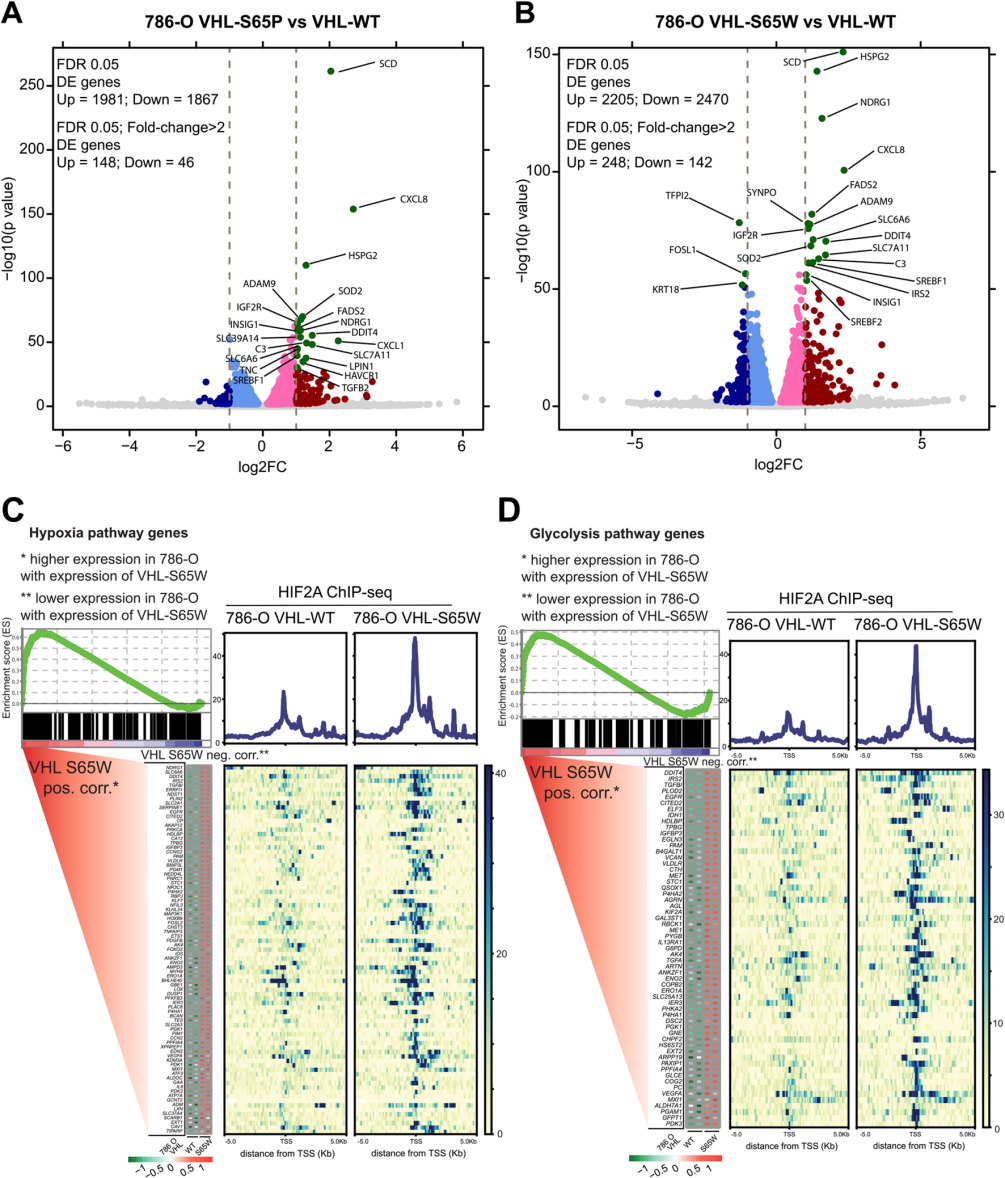
Gain of function of VHL mutations enhance hypoxia signaling and glycolysis pathways. **A**, **B** Volcano plots of differentially expressed genes in 786-O cells expressing VHL mutations, S65P (**A**) or S65W (**B**), compared with VHL WT control. Significantly altered genes (adjusted p value < 0.05; fold change >  ± 2) are indicated in green (upregulated) or dark blue (downregulated). Top ranked VHL mutation-influenced genes are selectively labeled. **C**, **D** GSEA-determined hypoxia (**C**) or glycolysis (**D**) pathways with S65W-influenced gene signature. Genes are ranked by their expression levels in 786-O cells expressing VHL mutation S65W. Plots in the right of C or D show HIF2α ChIP-seq signals of 5 kb around the transcriptional start sites (TSS) of altered genes in hypoxia (**C**) or glycolysis (**D**) pathways

**Fig. 5:**
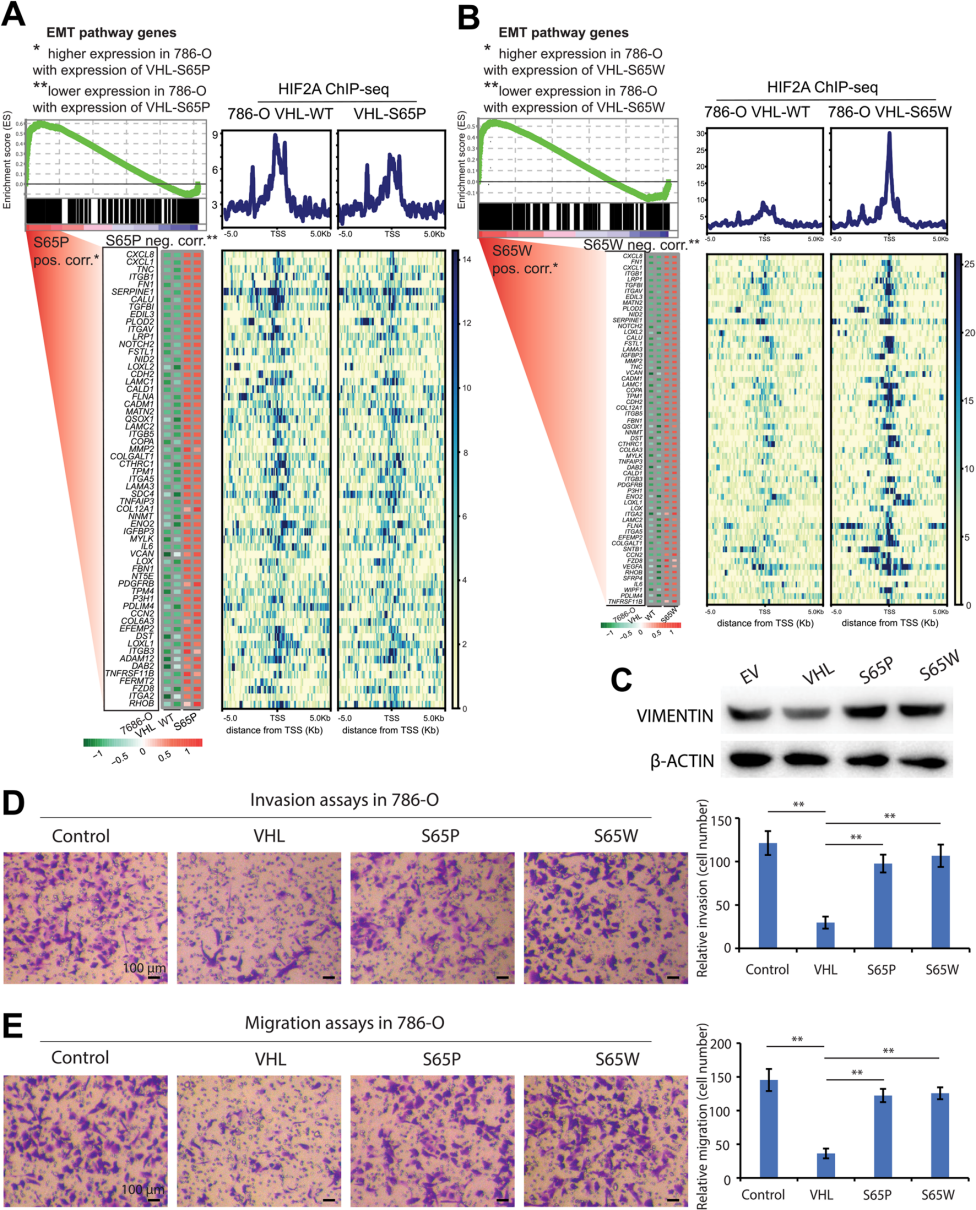
Gain of function of VHL mutation promotes epithelial-mesenchymal transition (EMT). **A**, **B** GSEA-determined EMT pathways with VHL S65P (**A**) or S65W-influenced gene signature (**B**). Genes are ranked by their transcriptional levels in S65P (**A**) or S65W (**B**) expressed 786-O cells. Right panels of A or B indicate HIF2α ChIP-seq signals of 5 kb around TSS of EMT pathway genes influenced by VHL S65P (**A**) or VHL S65W (**B**). **C** Western blot of the EMT marker VIMENTIN protein levels across 786-O cell lines expressing empty vector (EV), VHL WT or mutants. **D** Left panel: representative images of invasion assays for control 786-O cells or the 786-O cell lines with VHL or VHL mutant expression. Right panel: quantitation of relative invasion for 786-O cells. **E** Left panel: representative images of migration assays for control 786-O cells or the 786-O cell lines expressing VHL or VHL mutants. Right panel: quantitation of relative migration for 786-O cells. In D,E, error bars s.d. n = 3 technical replicates. P values were assessed via the two-tailed Student’s t test

To better delineate the roles of VHL mutants from wild type and investigate whether the impacts on HIF2α genomic binding differences are also reflected in gene expression profiles, we performed RNA sequencing (RNA-seq) analysis of functional target genes of HIF2α in the context of VHL genetic status. We observed a similar number of upregulated (n = 1981) and downregulated (n = 1867) by ectopic VHL-S65P expression (Fig. 4A), as well as 2,205 upregulated and 2,470 downregulated genes by ectopic VHL-S65W expression (Fig. 4B) in a default parameter (DEseq; FDR < 0.05) for differential expression (DE) analysis. While having stringent DE analysis (DEseq; FDR < 0.05, fold change > 2), we revealed a higher number of upregulated (n = 148) compared with downregulated genes (n = 46) under the expression of VHL-S65P, and upregulated (n = 248) compared with downregulated genes (n = 142) under the expression of VHL-S65W, suggestive of gain of function mutations in VHL stabilizing HIF2α. Among the most altered genes, it has been shown that renal cancer progression can be promoted by upregulation of CXCL8 [23], ADAM9 [24], NDRG1 [25], SOD2 [26], SREBF1 and SREBF2 genes [27]. The genes NDRG1 [25] and SREBF2 [27] have also been reported as potential prognostic biomarkers for RCC, and SREBF1/2 were found to play roles in regulating lipid metabolism and have effects on ccRCC growth and survival. To unravel the biological processes associated with these VHL mutant-regulated genes, we performed gene set enrichment analysis (GSEA). This bioinformatics analysis showed that hypoxia, glycolysis and EMT pathways were top enriched, in which genes upregulated by VHL mutations (Figs. 4C, D, 5A, B; Additional File 2: Fig. S2). In agreement with HIF2α ChIP-seq data as described above (Fig. 3F and G), compared to VHL-S65P expressing cells (Fig. 5A; Additional File 2: Fig. S2), HIF2α chromatin binding intensities are obviously stronger at these pathway genes in the ectopic VHL-S65W expression cells (Figs. 4C, D, 5B). Of the hypoxia signaling, known HIFα-regulated genes include ADM [28], VEGFA [12, 29], PDK3 [30], LOX [31], MXI1 [32], PDK1 [33], XPNPEP1 [34], SLC2A1 [34], IRS2 [35], and PFKFB3 [36], which have been shown to promote tumor growth and progression in various types of cancers. Previous reports showed that tumorigenesis is promoted via upregulation of glycolysis by ENO2 [37], PGK1 [38], ERO1A [39], P4HA1 [40], and IRS2 [41]. TGFBI is an extracellular matrix related oncogene that can lead to a significant enhancement of glycolysis for invasive tumor phenotype [42].

RCC is a leading cause of death for VHL disease-suffering patients and considered an epithelial malignancy [43, 44]. EMT has been reported to be important for RCC tumor progression and proposed as a potential mechanism of therapeutic resistance [45]. Remarkably, the EMT pathways are consistently found to be highly enriched in the 786-O cells expressing VHL-S65P or VHL-S65W mutations (Fig. 5A, B). Consistent with this, we observed an apparent upregulation of the mesenchymal marker, Vimentin (Fig. 5C), which is also increased in the RCC and CNSH specimens (Fig. 2D). In addition, we checked that expression levels of the EMT markers, *VIM* and *CDH1*, and found that *VIM* is markedly upregulated while *CDH1* is downregulated in KIRC tumors compared to normal kidney tissues (Additional file 2: Fig. S2C). We next performed cancer cell invasion and migration assays, and found that both the invasion and migration of 786-O cells stably expressing wild type VHL were significantly decreased compared to control cells with empty vector (Fig. 5D, E), consistent with accumulating evidence that VHL is a metastasis tumor suppressor of renal cancers [3]. By contrast, the 786-O cells with VHL-S65P or VHL-S65W expression exhibited apparent enhancement of invasion and migration compared to 786-O cells stably expressing normal VHL (Fig. 5D, E). Of the EMT pathway, the genes IL-6 [46], SFRP4 [47], FZD8 [48, 49], ADAM12 [50], and CCN2 [51] are known to promote cancer cell invasion and migration in multiple types of cancers. In addition, IGFBP3 in EMT pathway is a hypoxia-inducible gene and regulates EMT cellular processes while other genes indirectly promote EMT [52], e.g. CXCL8 inducing EMT through the PI3K/Akt/NF-κB signaling pathway [53]. Taken collectively, these results indicate potential clinical relevance of our findings that gain-of-functions in VHL mutations stabilize HIF2α protein and reprogram HIF2α genome-wide target genes.

### VHL mutant gene signature is associated with renal cancer progression and prognosis

**Fig. 6:**
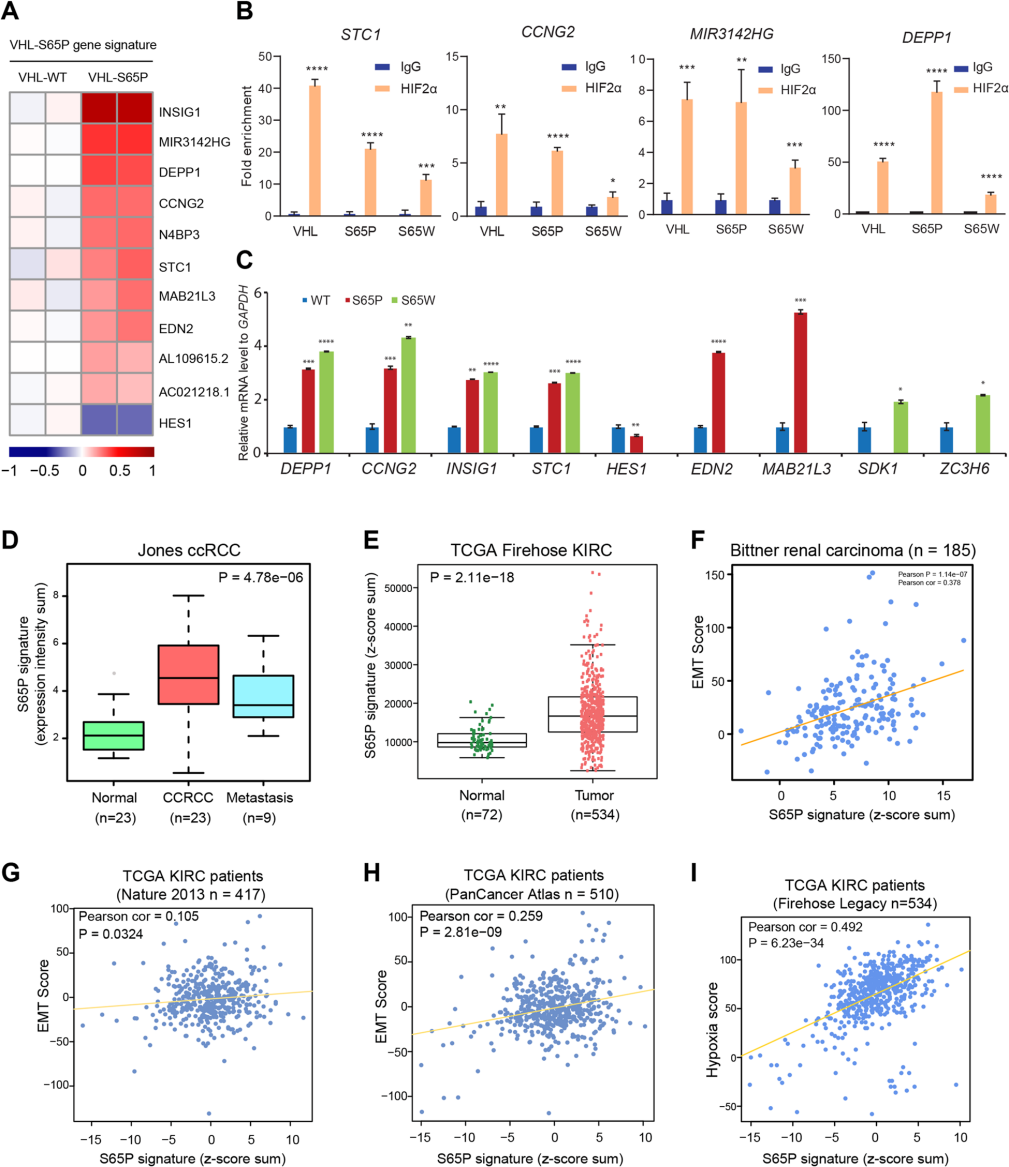
VHL-S65P gene signature is associated with human kidney cancer progression. **A** Heatmap showing the expression profiles of genes differentially altered by VHL-S65P and directly bound by HIF2α. **B** ChIP-qPCR confirmation of HIF2α chromatin binding at the representative genes of VHL mutant signature in the cells expressing WT or mutant VHL (n = 3 replicates). **C** Analysis of mRNA expression of the VHL mutant signature genes in 786-O cells expressing VHL WT or mutants using quantitative RT-PCR (n = 3). **D**–**E** VHL-S65P signature is significantly upregulated in human renal cancers compared to normal kidney samples. **F**–**I** The expression of VHL-S65 signature positively correlate with EMT score (**F**–**H**) and hypoxia signaling score (**I**)

To further address the clinical relevance of VHL mutations especially in human renal cancers, we performed an integrated analysis of HIF2α ChIP-seq data and VHL mutation-affected gene expression profiles, thereby identifying 11 genes directly bound by HIF2α and differentially altered by VHL-S65P. We defined this gene set as the VHL-S65P genetic signature (Fig. 6A). Representative genes from this gene signature were experimentally validated for chromatin occupancy of HIF2α through ChIP followed by real time quantitative PCR (Fig. 6B). Relative expression of the representative gene panel was also confirmed across VHL WT and S65P mutant expressing cells (Fig. 6C). By query from multiple clinical data sets [54–58], we observed that the expression levels of this VHL-S65P genetic signature were significantly higher in human renal carcinoma specimens than adjacent normal tissues (Fig. 6D, E and Additional File 3: Fig. S3). Given that VHL-S65P altered genes show an EMT pathway enrichment and enhanced hypoxia signaling, we asked whether there are any correlation between the VHL-S65P signature and the EMT or hypoxia score wherein higher values indicate more mesenchymal differentiation. This analysis revealed a consistent positive correlation of VHL-S65P signature with EMT score in the two cohorts of renal cancer patients (Fig. 6F–H), and hypoxia score in a cohort of patients with kidney cancer (Fig. 6I).

**Fig. 7:**
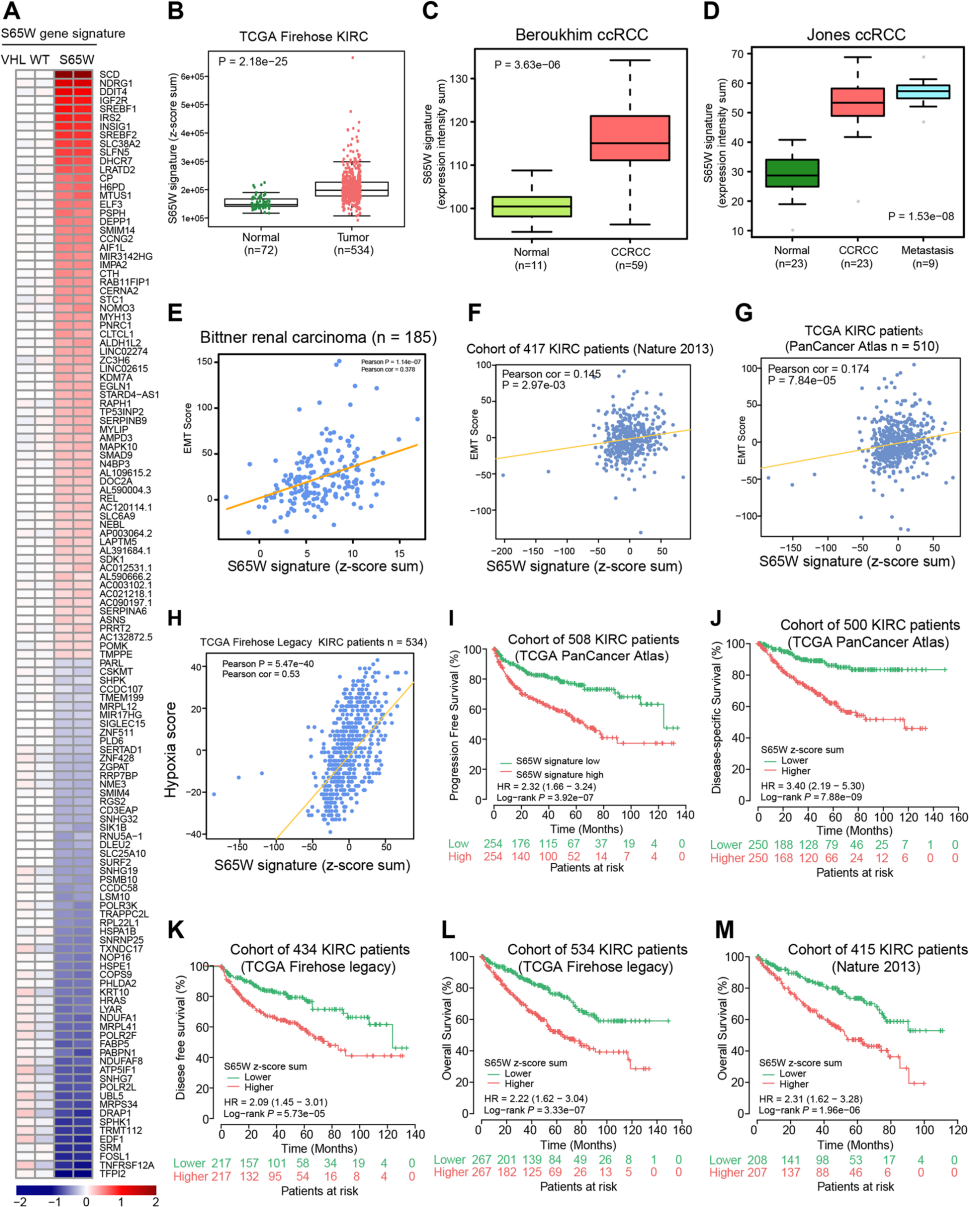
VHL-S65W gene signature is associated with human renal cancer severity and prognosis. **A** Heatmap showing the expression profiles of genes differentially influenced by VHL-S65W and directly occupied by HIF2α. **B**–**D** VHL-S65P signature is strikingly elevated in human kidney cancers compared to normal kidney specimens. **E**–**H** High expression of VHL-S65 signature positively correlate with elevated EMT (**E**–**G**) and hypoxia signaling score (**H**). **I** Kaplan–Meier graph showing a significant association between elevated expression of the VHL-S65W signature and shorter progression-free survival in a cohort of patients with renal cancers. **J**,** K** Kaplan–Meier graphs demonstrating significant associations between elevated VHL-S65W gene signature expression and reduced disease-specific survival (**J**) or disease-free survival (**K**). **L**, **M** Kaplan–Meier plots examining the risk of shortened overall survival in kidney cancer patients with higher expression of the VHL-S65W gene signature. In **I**–**M**, P values were calculated by a log-rank test

In a similar vein, we defined the VHL-S65W genetic signature with 128 genes directly occupied by HIF2α ChIP-seq and differentially expressed by VHL-S65W (Fig. 7A). HIF2α directed chromatin binding at representative genes and their expression across VHL WT and VHL S65W expressing cells were also validated (Fig. 6B, C). In the clinical settings, we observed that the S65W signature expression was significantly elevated in human kidney cancer specimens or metastatic sites in comparison with adjacent normal tissues [59] (Fig. 7B–D and Additional File 4: Fig. S4A-C), and positively correlated with the EMT scores [60] (Fig. 7E–G and Additional File 4: Fig. S4D,E) and hypoxia scores (Fig. 7H). To investiage whether the VHL-S65W signature possess clinical significance in KIRC patient prognosis, we performed a Kaplan–Meier survival analysis in several cohorts of human kidney cancer data sets. Strikingly, the results demonstrated that tumors with an elevated VHL-S65W signature showed reduced progression or disease-free survival as well as overall survival (Fig. 7I–M). Previous study has reported that knockout of the VHL gene led to EMT via upregulation of HIF1α regulated genes [61]. We have demonstrated differences in the effects on HIF2α functions between S65P and S65W. To further investigate whether there might be common or different impact between VHL deficiency and VHL-S65P/W mutations, we compared the RNA-seq profiling of the VHL deficiency and VHL-S65P/W mutations in the 786-O cells and performed Gene set enrichment analysis (GSEA) to identify functional pathways significantly enriched in up- or downregulated genes in each condition in the Hallmark gene sets. We found numerous common signatures enriched in up- or downregulated genes between VHL-deficiency and VHL-S65P/W (Additional file 5: Fig S5A, B), suggesting common impacts from VHL-deficiency and VHL-S65P/W mutations. In addition, we also found several signatures that are distinct between VHL-deficiency and VHL-S65P/W (Additional file 5: Fig S5C, D), further supporting that the underlying pathogenic mechanisms were not exactly the same between VHL-S65P and VHL-S65W. Altogether, these analyses further strengthen the clinical relevance and significance of the missense mutations in *VHL* gene in the familial von Hippel-Lindau disease, as described above.

## Discussion

More than 1000 families suffering VHL disease and over 650 kinds of germline *VHL* gene mutations have been documented in the Human Gene Mutation Database (HGMD) and the UMD-VHL mutations database. Our previous study of 540 patients from 187 unrelated Chinese families revealed that the high-frequency mutations included Ser65 (4.81%), Arg161 (5.35%), and Arg167 (12.84%) [62]. The results were different from previous studies in the Western and Japanese populations, suggesting that the spectrum of *VHL* gene mutations varies among populations with different ethnic backgrounds. In this study, we reported a missense mutation c.193T > C (p.Ser65Pro) that, for the first time, was detected in Chinese population. The c.193T > C mutation has previously been reported in the French VHL database [63] and a sporadic RCC cohort [64], and ClinVar contains an entry for this variant (Variation ID: 547829). The c.194C > G (p.Ser65Trp) mutation, a high frequency of mutation, has been reported in dozens of families worldwide, as well as our previous study [65], and ClinVar also contains an entry for this variant (Variation ID: 43,597).

The genotype–phenotype correlations are always research hotspots for VHL disease. The typical genotype–phenotype correlation is that VHL missense mutations are responsible for type 2 disease with a high risk of PHE, and truncating mutations are responsible for type 1 disease with a low risk of PHE [66]. Our previous study has yielded similar results, and we demonstrated that ﻿*VHL* missense mutations in the HIFα binding site residues 65–117 increased the risk of age-related CNSH and RCC [65]. In this study, the patients of Family 1, the proband (III-1) and his father (II-1), were classified as type 1 VHL disease (without PHE), but there was no data about the phenotype of the French family mentioned above [63]. The patients of Family 2 were classified as type 1 VHL disease (without PHE), consistent with other families with the c.194C > G mutation reported previously. These results emphasize the genotype–phenotype correlations based on the alteration of a HIFα binding site in the pVHL.

Normal pVHL is crucial for HIFα (including HIF1α or HIF2α) ubiquitylation and subsequent proteolytic degradation in normal oxygen concentration. *VHL* mutations often result in pVHL dysfunction and HIFα accumulation. Despite of the controversy, HIF1α and HIF2α have been identified to be essential for VHL disease, particularly for ccRCC and hemangioblastoma. Over the past decades, emerging evidence revealed that HIF2α, rather than HIF1α, is the key driver of RCC progression [14]. However, more recent studies have suggested the significance of HIF1α during tumor initiation and development [67]. A review of various immunohistochemical studies demonstrated that HIF1α protein is detectable in approximately 70% of ccRCCs [68], which are consistent with our IHC staining results of RCC and hemangioblastoma tissues.

In our study, the c.193T > C mutation causes Serine to Proline substitution at residue 65 (p.Ser65Pro or VHL-S65P), and c.194C > G mutation replaces Serine with Tryptophan (p.Ser65Trp or VHL-S65W). The serine residue (Ser65) and its nearby residues are highly evolutionarily conserved among different species, suggesting its importance for maintaining the protein’s structure and function. Furthermore, Ser65 is a hotspot mutation in Chinese populations [62] and other mutations leading to Ser65 substitution, including c.193T > G (p.Ser65Ala), c.194C > A (p.Ser65Ter), c.194C > T (p.Ser65Leu), have been documented in ClinVar, HGMD and the UMD-VHL mutations database. This suggests that Ser65 is clinically significant, and those variants that disrupt this residue are likely to be disease-causing. VHL-S65W mutation has been found in several families with VHL disease as our previous studies presented [62, 65], while VHL-S65P was rarely reported and for the first time discovered in a Chinese family in present study. Intriguingly, we found that VHL-S65P and VHL-S65W are sensitive to the ubiquitin–proteasome system mediated degradation, consistent with the work showing *VHL* loss can stabilize HIF2α [69], thereby mechanistically explaining the accumulation of HIF2α observed in our current study. In addition, Ser65 is located in the β-domain of pVHL and within the HIFα binding sites. Felicia Miller et al. [20] revealed that VHL-S65W was deficient in both targeting HIF2α for degradation and binding to HIF1α, implying that HIF2α accumulation was also the results of deficient binding capabilities of VHL-S65P and VHL-S65W.

By ChIP-seq assays on genome-wide HIF2α chromatin binding, we further demonstrated that the *VHL* mutations influence not only HIF2α global chromatin binding profile but also its transcriptional regulatory activities. We also found that an overall slightly decreased chromatin intensity for HIF2α in VHL-S65P cells, and strongly increased intensity in VHL-S65W cells. Consistently, RNA-seq analysis revealed that *VHL* mutations dramatically upregulated a number of genes, especially in the pathways of hypoxia, glycolysis and EMT, and this enhanced transcriptional effect was stronger in VHL-S65W cells than VHL-S65P cells. These observations indicated that the underlying pathogenic mechanisms were not exactly the same between VHL-S65P and VHL-S65W. Through functional study and clinical relevance analysis, we further demonstrated that gain-of-functions in VHL-S65P/S65W stabilize HIF2α protein and reprogram HIF2α genome-wide target genes, and emphasize the clinical relevance and significance of the missense mutations of *VHL* gene in the VHL disease-associated tumors. These results emphasize the significance of Ser65 residue in pVHL function and strengthen the crucial role of pVHL-HIF dysregulation in VHL disease, particularly for RCC.

## Conclusions

In summary, our findings highlight the crucial role of pVHL-HIF dysregulation in VHL disease and strengthen the clinical relevance and significance of the missense mutations of Ser65 residue in pVHL in the familial VHL disease. Our results provide strong support for the pathogenicity of the *VHL* c.193T > C (p.Ser65Pro) and c.194C > G (p.Ser65Trp) mutations, which are important supplementary data for evaluating these mutations as “pathogenic” (variation ID: 547,829, 43,597) in ClinVar of NCBI, and shows an example of documenting the mechanistic effects of the other types of VHL mutations on disease progression.

## Methods

### DNA extraction and sequence analysis

Genomic DNA was isolated from peripheral blood using a TIANamp Blood DNA Kit (Tiangen, Beijing, China). After polymerase chain reaction (PCR) amplification, Sanger DNA sequencing was performed. DNA sequences were analyzed with a genomic reference sequence on NCBI BLAST (NM_000551.3). The mutation was named according to the Human Genomic Variation Society (HGVS: http://www.hgvs.org/). All primers were listed in Additional file 7: Table S2.

### Histology and Immunohistochemical staining

Renal and brain tumors were surgically removed from the proband (III-1) and postoperatively assessed for pathological confirmation. Sections of these tumors were fixed and stained with hematoxylin and eosin (H&E) for morphological evaluation. The primary antibodies were listed: anti-pVHL (Cell Signaling Technology, CST, MA, USA), anti-HIF1α (CST), anti-HIF2α (R&D Systems, MN, USA), anti-E-cadherin (CST) and anti-Vimentin (CST). The immunostaining images were captured using Olympus FSX100 microscope (Olympus, Tokyo, Japan).

### Cell lines and cell culture

The human ccRCC 786-O cell line was purchased from the CLS Cell Lines Service (Eppelheim, Germany). The 786-O cells were cultured at 37 °C, with RPMI 1640 medium (Sigma) supplemented with 10% fetal bovine serum (Gibco, Australia origin, Grand Island, NY, USA) and 1% of Penicillin/Streptomycin (Thermo Fisher Scientific, USA). For chemical hypoxia mimetic experiments, cells were grown in the presence or absence of CoCl_2_ (Sigma-Aldrich) for 6 h and DMOG (Sigma-Aldrich) for 8 h.

### Western blot analysis

Proteins extracted by RIPA lysis buffer from cell lines were fully electrophoresed with 10% SDS-PAGE gel and transferred to PVDF membrane. Then, the membranes were blocked with 5% Albumin from bovine serum (BSA) and incubated in primary antibodies against targeted protein at 4 °C overnight. After a further incubation with HRP-conjugated secondary antibody, the bands were scanned and processed as supplier’s instruction. The primary antibodies that we used included: anti-FLAG (Sigma, USA), anti-VHL, anti-HIF1α, anti-HIF2α (Novus Biologicals) and anti-ACTIN (CST).

### Lentiviral constructs, lentivirus production, and infection

The wild type (WT) VHL was cloned into the Tet-one vector with FLAG-tag, VHL point mutations in the FLAG-VHL vectors were generated by site-directed mutagenesis and verified by DNA sequencing. To generate the FLAG-VHL 786-O overexpression cell lines, third-generation lentiviral vectors were packaged using 293 T cell line. In brief, the 293 T cells were seeded on 10-cm plates until cells reach 60%–80% confluency. Lipofectamine 2000 was applied for the transfection, cells were co-transfected with indicated FLAG-VHL overexpression constructs (6 µg each), 2 µg each of pVSVG (envelope plasmid), pRSV-Rev (packaging plasmid), and pMDLg/pRRE (packaging plasmid). The culture medium was replaced with fresh medium after 24 h transfection. The lentivirus was collected after 48 h transfection. For the viral transduction, 786-O cells were plated in 6-well plate until cells reach 60–70% confluency. The medium was replaced with indicated lentivirus-containing medium and incubated for 24 h. 2 µg /ml puromycin (Sigma) was used for the selection of the transfected cells. After successfully obtained the transfected FLAG-VHL 786-O cell lines, the overexpression of FLAG-VHL were induced by 1 µg /ml doxycycline.

### Invasion and migration assays

Cell invasion and migration were performed using 8 μm pore size 24-well Transwell inserts (Corning Costar) with or without Matrigel (20 μg/filter) (Corning). 786-O cells were pre-treated with 1 μg/ml doxycycline one day before. Cells were detached and resuspended into serum-free medium. 200 μl serum-free medium with 10^4^ cells and 1 μg/ml doxycycline were plated into the inserts. The lower chambers were filled with 700 μl complete medium with 1 μg/ml doxycycline. After 20 h, the cells were fixed with 3.7% formaldehyde and stained with 0.1% crystal violet solution. The inner surfaces of the filters were cleaned by cotton swab. The invasive and migrated cells were quantified by counting the cells on the lower side of the filters under a light microscope. The results obtained from three replicate inserts, and statistical analyses were performed by two-tailed Student’s t test.

### Immunofluorescence

For immunofluorescence staining, 786-O cells were plated onto gelatin-coated cover glasses in a 12-well plate. Cells were fixed and stained with antibodies recognizing FLAG-VHL (Sigma, F1804, 1:500).

### Co-Immunoprecipitation (Co-IP)

Co-Immunoprecipitation was performed for examining the interaction of FLAG-VHL with HIF2α in 786-O cells. The 786-O cells were harvested and lysed with 0.5 ml cold immunoprecipitation buffer (50 mM Tris–HCl, pH 7.5, 1 mM EDTA, 150 mM NaCl, 10% Glycerol, 1% Triton X-100 and cOmplete protease inhibitor cocktail (Roche)). The cell lysates were incubated on ice for 30 min followed 20 s sonication. After 30 min centrifugation at 4 °C, transfer the supernatant to a new tube and incubated with 30 μl Dynabead protein G (Invitrogen) at 4 °C for 2 h, to pre-clear crude cell extract of proteins. Transfer the supernatant and add 5-8 μg of FLAG antibody incubate at 4 °C overnight. Add 30 μl of protein-G beads and incubate at 4 °C for 6 h followed by washing three times with Immunoprecipitation buffer. Resuspend the beads in 30 μl of 2 × SDS sample loading buffer and incubate at 95 °C for 5 min. The supernatants were used on SDS-PAGE gel for electrophoresis separation.

### Quantitative real-time polymerase chain reaction (qRT-PCR)

Total RNA was isolated from cultured cells via RNeasy Mini Kit (QIAGEN), and DNA was removed by RNase-Free DNase (QIAGEN) in these samples. cDNA was synthesized using the High-Capacity cDNA Reverse Transcription Kit (Thermo Fisher Scientific, USA). A qRT-PCR analysis was performed with the SYBR Select Master Mix (Applied Biosystems). For ChIP-qPCR experiments, all the data were normalized to the control regions, the relative enrichment of the target antibodies at target DNA fragment were determined by compared with the IgG control. The associated primers were listed in Additional file 7: Table S2.

### Chromatin immunoprecipitation sequencing (ChIP-seq)

786-O cells were cross-linked with 1% formaldehyde for 10 min, followed by 5 min 125 mM glycine treatment to stop the reaction. Cells were washed twice by cold PBS and collected as pellets. Cell pellets suspended in hypotonic lysis buffer (10 mM KCl, 20 mM Tris–Cl, PH 8.0, 2 mM DTT, 10% glycerol and cOmplete protease inhibitor cocktail (Roche)) up to 50 min to isolate nuclei. The nuclei pellets were resuspended in 1:1 ratio of ChIP dilution buffer (0.01% SDS, 1.1% Triton X-100, 16.7 mM Tris–HCl, pH 8.1, 1.2 mM EDTA, 167 mM NaCl and cOmplete Protease Inhibitor) and SDS lysis buffer (1% SDS, 50 mM Tris–HCl, pH 8.1, 10 mM EDTA, and cOmplete Protease Inhibitor). The chromatin was sonicated by Q800R sonicator (QSonica) to generate an average size of 300 bp fragments at 4 °C. Blocking buffer ((0.5% BSA in IP buffer (20 mM Tris–HCl, pH8.0, 150 mM NaCl, 1% Triton X-100, 2 mM EDTA, and cOmplete protease inhibitor cocktail (Roche)) pre-washed Dynabead protein G (Invitrogen) was incubated with antibody against HIF2α (AF2886, R&D Systems). The fragmented chromatin lysate was precipitated with antibodies/beads complexes at 4 °C for 12 h. The complex was washed 4 times with RIPA washing buffer (0.5 M LiCl, 50 mM HEPES, PH 7.6, 1 mM EDTA, 1% NP-40 and 0.7% sodium deoxycholate), and 2 times with 100 mM ammonium hydrogen carbonate (AMBIC) solution. Extraction buffer (1% SDS, 10 mM Tris–HCl, pH8.0 and 1 mM EDTA) was used to separate the DNA–protein complexes from the beads and incubated with NaCl and Proteinase K to reverse the cross-links at 65 °C overnight. The ChIP DNA was extracted by Mini-Elute PCR purification kit (Qiagen) and subjected to library preparation by NEBNext® Ultra™ II DNA Library Prep Kit (E7103L, NEB). A NovaSeq 6000 S4 sequencing system (Illumina, San Diego, CA, USA) was used to sequence the DNA libraries.

### RNA sequencing

Total RNA was extracted from 786-O cells overexpressing either WT or mutant VHL and control cells expressing empty vector each with two replicates. The quality and quantity of total RNA was measured by Agilent Bioanalyzer 2100 (Agilent Technologies) with Eukaryote Total RNA Nano Kit (Agilent Technologies), and NanoDrop Spectrophotometer (Thermo Scientific). NEBNext® Ultra™ RNA Library Prep Kit (Illumina) was used according to the manufacturer's instructions. Bioanalyzer 2100, Qubit 2.0 Fluorometer and qPCR were used for the assessment of quantification and quality of libraries. A NovaSeq 6000 S4 sequencing system (Illumina) was used to sequence the RNA libraries (paired end 150 bp).

### Bioinformatics analysis of ChIP-seq data

ChIP-seq libraries of HIF2A (786-O cells expressing VHL WT or VHL S65 mutations) were sequenced to generate 150 bp single-end reads. Sequence quality of raw data was assessed by FastQC [70] and Trimmomatic [71] was applied for quality control with parameters TruSeq3-SE.fa:2:30:10 SLIDINGWINDOW:5:20 MINLEN:30. The processed ChIP-seq data were aligned to the human hg38 genome assembly with Bowtie2 [72]. The ChIP-seq peaks were called by MACS2 [73] with parameter “-q 0.05 –keep-dup 1”, while all other parameters remained as default. DNA Motif enrichment analysis was performed using SeqPos [74]. Common and unique peaks between VHL WT and mutations were generated by bedtools v2.29.0. ChIP-seq peaks were annotated by (HOMER v4.10) [75] “annotatePeaks.pl”. Bioconductor package ChIPseeker (1.18.0) [76] was applied to annotate and visualize genomic location of peaks from WT, S65P and S65W in terms of genomic features. Bam files were first converted into bigWig files by using bamCoverage from deepTools2. Heatmaps of aligned reads and average signal plots were generated by SAMtools (v1.9) [77] and deepTools2 (v3.3.2) [78].

### RNA-seq data processing and differential gene expression analysis

For the RNA sequencing of 786-O cells expressing VHL WT or VHL S65 mutations, paired-end raw sequence reads were first pre-processed with FastQC [70] to assess the read quality. Trimmomatic [71] was employed to process reads for quality trimming and adapter removal with parameters TruSeq3-PE-2.fa:2:30:10 SLIDINGWINDOW:5:20 MINLEN:30. A final FastQC run was performed to ensure the success of previous quality control steps. The processed reads (FASTQ files) were mapped to the human genome reference sequence hg38/GRC38 using STAR version 2.7.2a [79] with default settings. Quantification of gene expression was performed via HTSeq (htseq-count) with parameters “–r pos -s no -i gene_name”, GENCODE v33 primary assembly was used as reference gene-annotation set. Differential gene expression analysis was performed from read count matrix generated from HTSeq by employing Bioconductor package DESeq2 (1.26.0) [80]. Genes with low counts (< 2 cumulative read count across samples) were filtered out prior to downstream analysis. Differentially expressed genes were called with cutoff FDR < 0.05. Method Variance Stabilizing Transformation (VST) from DESeq2 was applied for data normalization. The expression concordance among biological replicates was measured by a sample-to-sample distance matrix using hierarchical clustering with Euclidian distance metric from normalized total transcriptome of each sample. Heatmaps displaying differentially expressed genes between treatment and control samples were generated using R package “pheatmap” (1.0.12).

### Gene set enrichment analysis

Gene Set Enrichment Analysis (GSEA) [81] was performed using the Preranked method using Hallmark gene sets. Genes were ordered in a descending order based on the T-statistic from the differential expression analysis via DESeq2. Parameters were set as follows: Enrichment statistic = “weighted”, Max size (exclude larger sets) = 5000, number of permutations = 1000. All other parameters were left as default. The GSEA enrichment plots were generated using R packages “clusterProfiler” (3.14.3) [82] and “enrichplot” (1.6.1) [83].

### Development of the VHL S65P and S65W signatures

We defined the VHL S65P and S65W signatures by first selecting a list of differentially expressed genes between VHL S65P or S65W mutation and VHL WT control in the 786-O cell line with the cutoff FDR < 0.05 and log2 (fold change) ≥ 1 or ≤ − 1. We next determined the overlap of these genes with corresponding HIF2A genome-wide chromatin binding sites from the 786-O cell line harboring VHL S65P or S65W mutations. We finally devised the VHL-S65P (11 genes) or S65W (128 genes) direct target gene signatures that displayed both HIF2A chromatin binding in the promoter (TSS ± 5 kb) as well as dysregulation upon RNA-seq profiling of VHL S65P or S65W mutation in 786-O cells. S65P and S65W signature scores were calculated as weighted sums of normalized expression of the genes in the signatures and Pearson correlation test was performed to compare with EMT, Hypoxia scores to test the associations.

### EMT score, hypoxia score and the EMT pathway genes

The EMT score, consisting of 76 genes, was referenced from a study conducted by Bayer et al. [84], from which the EMT signature was found correlated with known EMT markers. The hypoxia score used in this study was obtained from a study carried out by Bhandari et al. [85]. The authors quantified tumor hypoxia in 8,006 tumors from 19 tumor types in The Cancer Genome Atlas (TCGA) and the Canadian Prostate Cancer Genome Network (CPC-GENE) by using mRNA-based hypoxia signatures from eight independent studies. The hypoxia scores were proved highly consisted in the prediction of hypoxia for primary tumours. The EMT pathway genes, consisting of 200 genes, were retrieved from the Hallmark gene sets from the MSigDB database [81].

### Statistical analysis

All statistical analyses were performed using R (v. 3.6.3) with RStudio (v. 1.2.5033). Data were obtained from the cBioPortal for Cancer Genomics [86, 87] and published literature. Differential gene expression analyses were applied between normal prostate and tumor tissues from various independent cohorts. Statistical analyses were performed to study the correlation between gene expression levels and clinical features including Tumor stage, Lymph nodes, Metastasis stage and Neoplasm disease stage. Statistical tests for patients stratified into two or more groups were calculated using the Mann–Whitney U test and Kruskal–Wallis H test, respectively. Kaplan Meier survival analysis from R package “Survival” (v. 3.2.3) [88] and “Survminer” (v. 0.4.7) [89] was used to assess the impact of VHL-S65P or VHL-S65W mutation on kidney renal clear cell carcinoma (KIRC) [90, 91] prognosis. Patients were stratified according to median expression of the weighted sum of the signature. Function “Surv” was first used to generate the survival models with “time-to-event” and “event status” as input from clinical cohorts. Median expression of genes was further followed to fit to the models by function “survfit”. Statistical analyses for all Kaplan Meier curves were calculated using log-rank test. Cox proportional hazards model was applied to evaluate the hazard ratio (HR). Samples with missing expression or patient survival data were excluded from analyses. *P* value < 0.05 was considered to be statistically significant.

## Supplementary Information

**Additional file 1: Figure S1**. (A) Quantitative of HIF-2α expression levels in 786-O cells stably expressing VHL WT and mutants, with or without treatment of MG132. The western blot images were scanning densitometric values were obtained using ImageJ software. HIF-2α Protein levels were normalized to the loading control β-ACTIN. (B) Interaction of HIF-2α with VHL-WT and mutants after MG132 treatment. (C) Western blot for HIF-2α expression in 786-O cells treated with or without hypoxia mimetic CoCl2 and DMOG. CoCl2 was used at 100 uM final concentration for 6 h, DMOG was used at 1 mM final concentration for 8 h. (D) Immunostaining of FLAG-VHL in 786-O cells with and without MG132 treatment, Scale bars, 50 μm.

**Additional file 2: Figure S2**. (A-B) GSEA-determined hypoxia (A) or glycolysis (B) pathways with S65P-affected gene signature. Genes are ranked by their expression levels in 786-O cells expressing VHL mutation S65P. Plots in the right of A or B show HIF2α ChIP-seq signals of 5 kb around the transcriptional start sites (TSS) of altered genes in hypoxia (A) or glycolysis (B) pathways. (C) VIM or CDH1 is upregulated or downregulated in KIRC patient samples, respectively.

**Additional file 3: Figure S3**. (A-D) The sum expression levels of VHL-S65P signature gene set are significantly upregulated in human renal cancers compared to normal kidney samples.

**Additional file 4: Figure S4**. (A-C) The sum expression levels of VHL-S65W signature gene set are significantly upregulated in human renal cancers compared to normal kidney samples. (D,E) The sum expression levels of VHL-S65W genetic signature positively correlate with EMT scores in the two independent clinical cohorts of ccRCC patient group.

**Additional file 5: Figure S5**. Gene set enrichment analysis (GSEA) analysis identifies significant common (A and B) and distinct Hallmark signatures between VHL-deficiency and VHL-S65P/W (C and D). Gene signatures with FDR <0.05 are considered as significant

**Additional file 6: Table S1**. The statistical results of VHL mutations S65P and S65W patients.

**Additional file 7: Table S2**. Primers sequences.

## Data Availability

The authors declare that all data and materials are available on request. All raw ChIP-seq and RNA-seq data have been deposited in the European Nucleotide Archive (ENA) under accession ID PRJEB52303 and PRJEB52308, respectivily.

## Abbreviations

VHL: Von Hippel-Lindau
Ser: Serine
Pro: Proline
Trp: Tryptophan
HIFα: Hypoxia-inducing factor α
RH: Retinal hemangioblastoma
CNSH: Central nervous system hemangioblastoma
RCC: Renal cell carcinoma
PHE: Pheochromocytoma
PCT: Pancreatic cysts or tumors
qRT-PCR: Quantitative real-time PCR
ChIP-seq: Chromatin Immunoprecipitation Sequencing
CoCl_2_: Cobalt chloride
DMOG: Dimethyloxallyl Glycine
GSEA: Gene Set Enrichment Analysis
MRI: Magnetic resonance imaging
