# Supplementary figures and images for: VHL Ser65 mutations enhance HIF2α signaling and promote epithelial-mesenchymal transition of renal cancer cells
Source: Cell Biosci. 2022 May 3;12:52. doi: 10.1186/s13578-022-00790-x (PMC9066845; doi:10.1186/s13578-022-00790-x)

Additional file 1

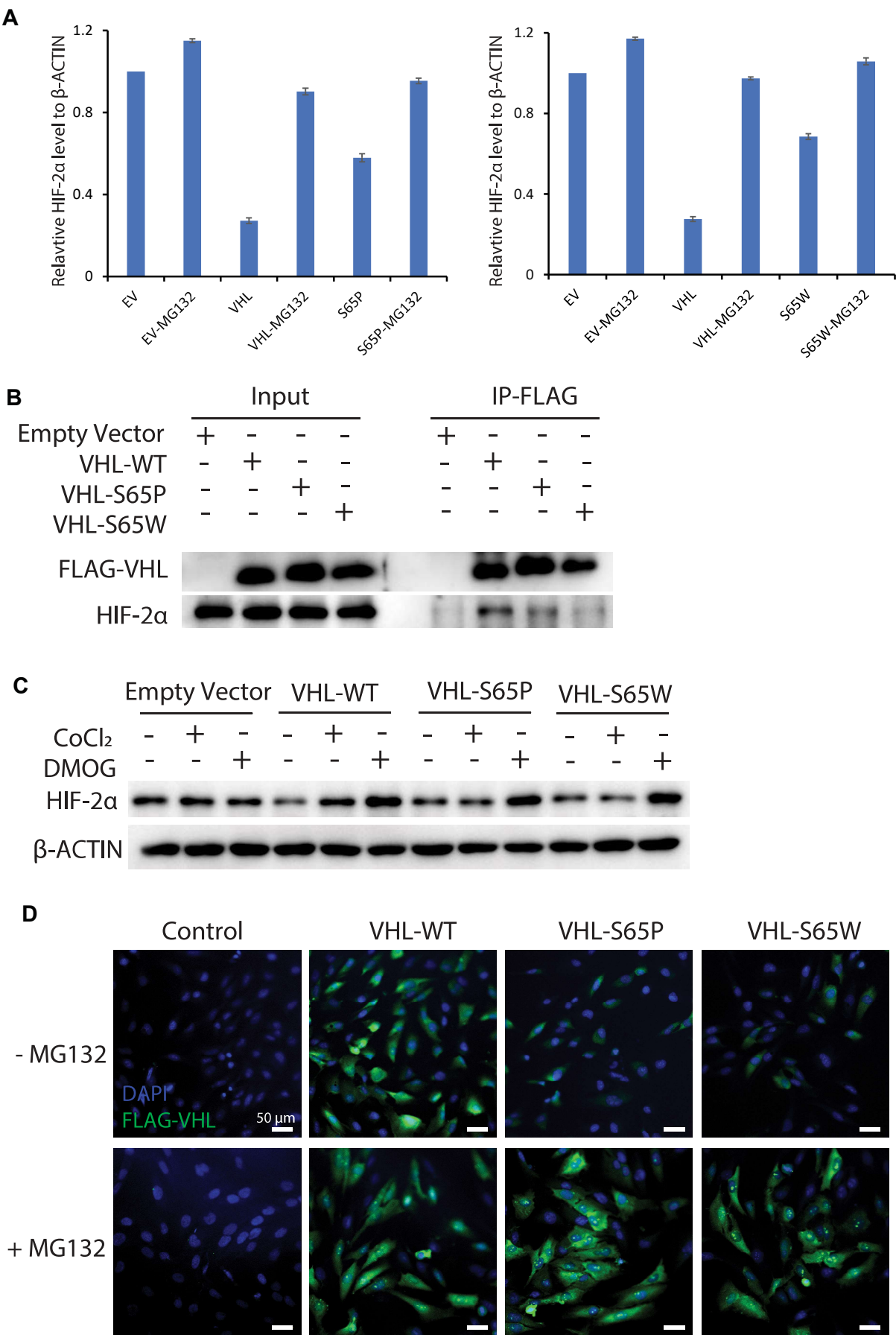

Supplement: Supplementary file 1 — Additional file 1: Figure S1. (A) Quantitative of HIF-2α expression levels in 786-O cells stably expressing VHL WT and mutants, with or without treatment of MG132. The western blot images were scanning densitometric values were obtained using ImageJ software. HIF-2α Protein levels were normalized to the loading control β-ACTIN. (B) Interaction of HIF-2α with VHL-WT and mutants after MG132 treatment. (C) Western blot for HIF-2α expression in 786-O cells treated with or without hypoxia mimetic CoCl2 and DMOG. CoCl2 was used at 100 uM final concentration for 6 h, DMOG was used at 1 mM final concentration for 8 h. (D) Immunostaining of FLAG-VHL in 786-O cells with and without MG132 treatment, Scale bars, 50 μm. [file 13578_2022_790_MOESM1_ESM.pdf]

Additional file 2

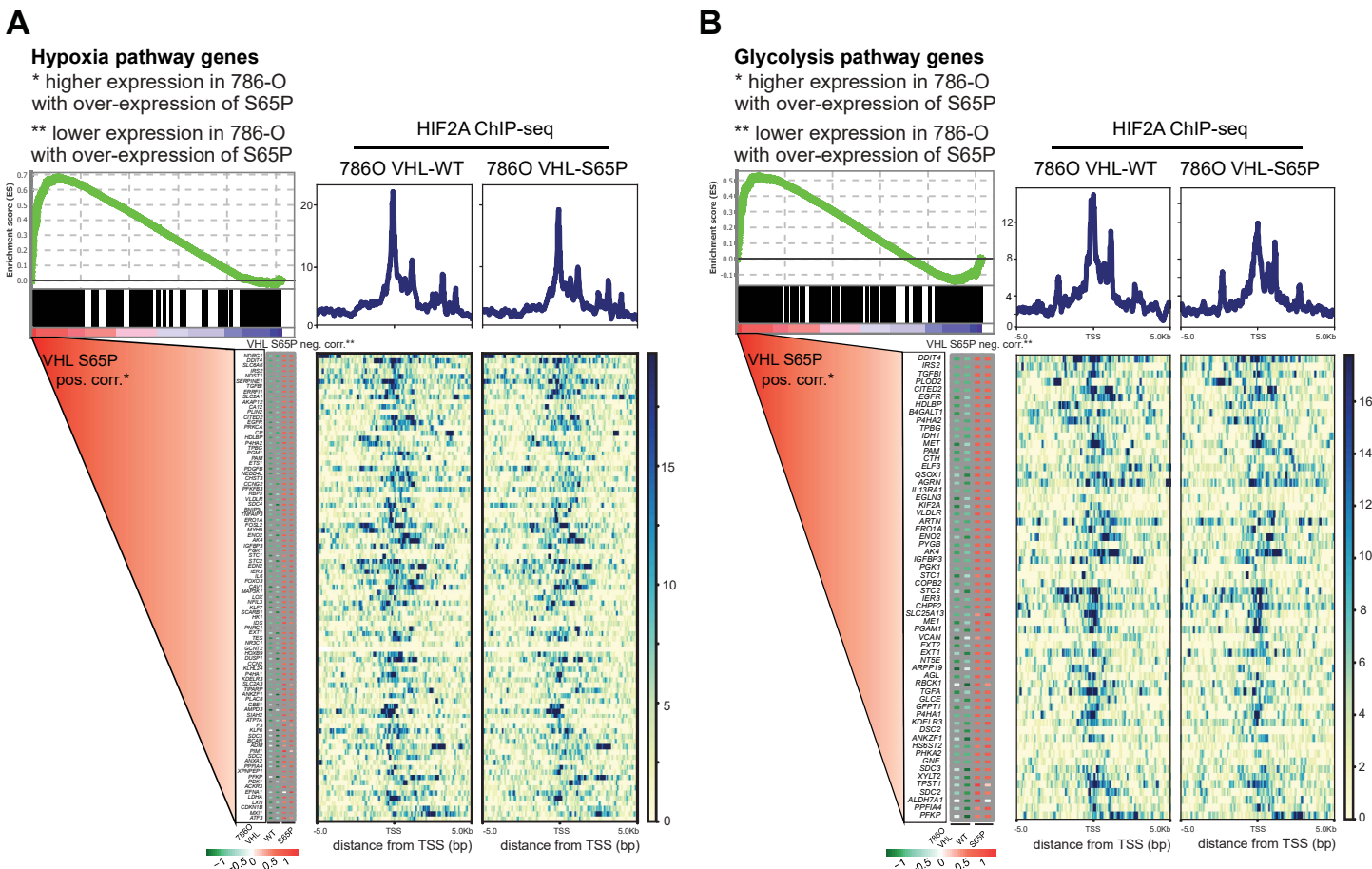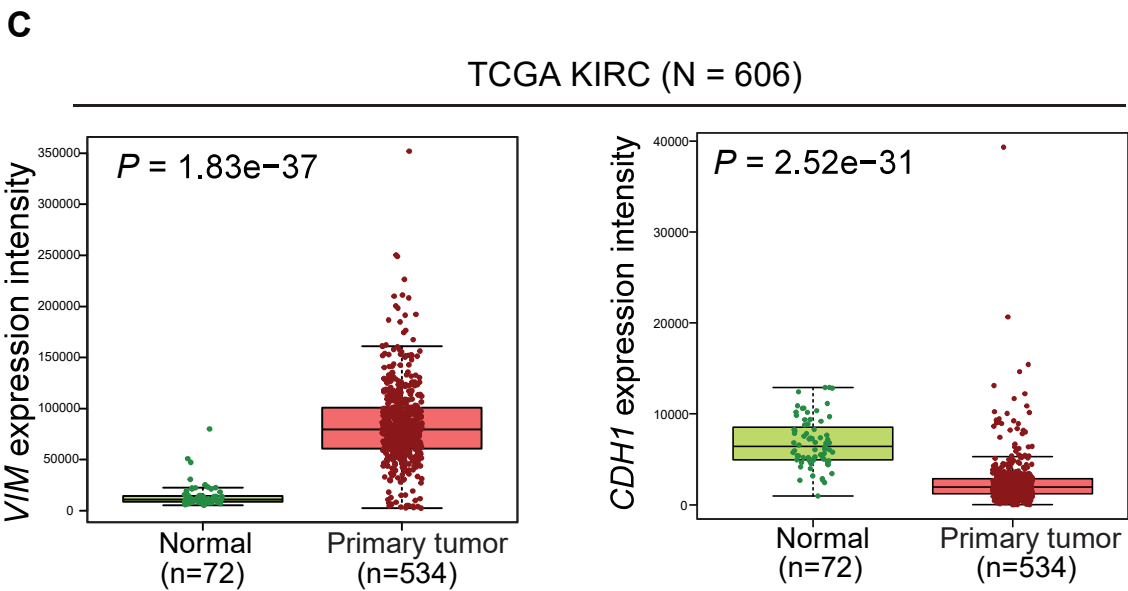

Supplement: Supplementary file 2 — Additional file 2: Figure S2. (A-B) GSEA-determined hypoxia (A) or glycolysis (B) pathways with S65P-affected gene signature. Genes are ranked by their expression levels in 786-O cells expressing VHL mutation S65P. Plots in the right of A or B show HIF2α ChIP-seq signals of 5 kb around the transcriptional start sites (TSS) of altered genes in hypoxia (A) or glycolysis (B) pathways. (C) VIM or CDH1 is upregulated or downregulated in KIRC patient samples, respectively. [file 13578_2022_790_MOESM2_ESM.pdf]

Additional file 3

**A**

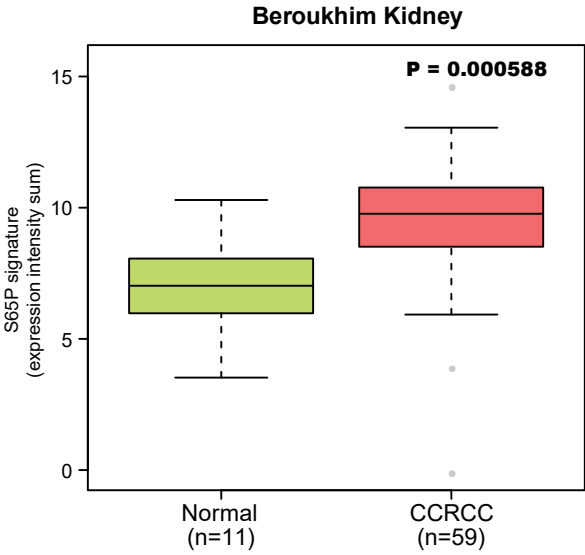

**B**

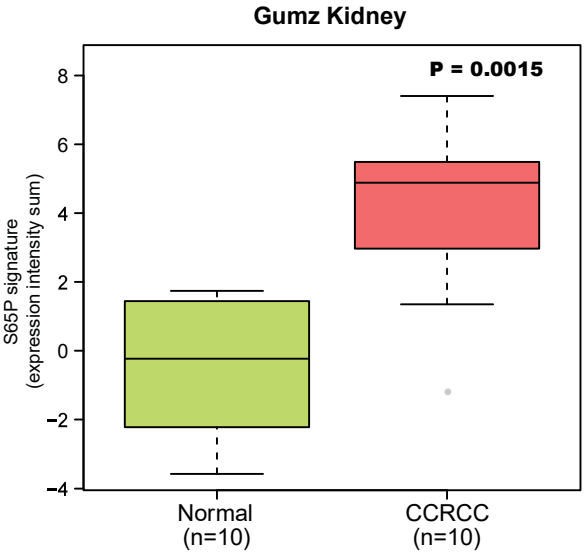

**C**

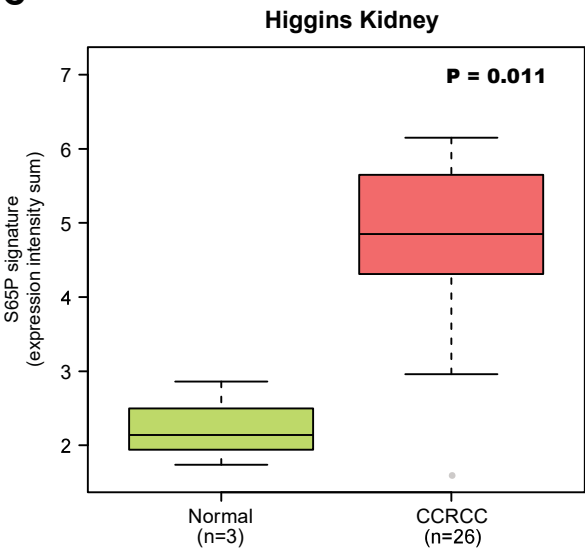

**D**

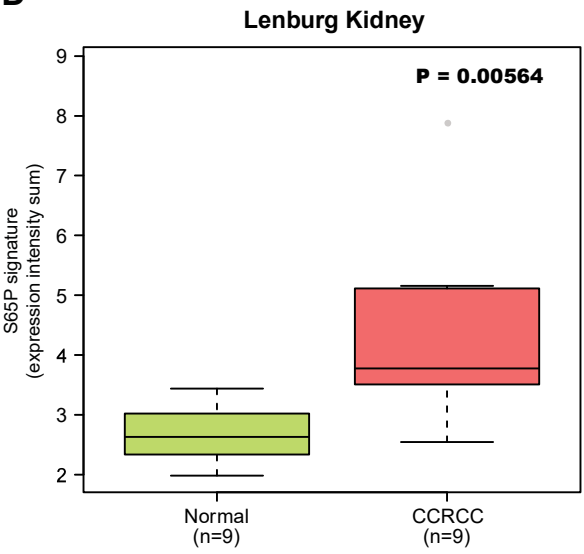

Supplement: Supplementary file 3 — Additional file 3: Figure S3. (A-D) The sum expression levels of VHL-S65P signature gene set are significantly upregulated in human renal cancers compared to normal kidney samples. [file 13578_2022_790_MOESM3_ESM.pdf]

## Additional file 4

**A**

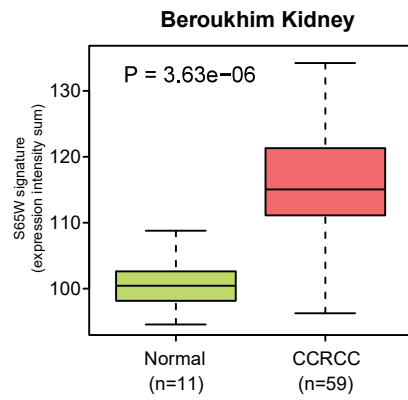

**B**

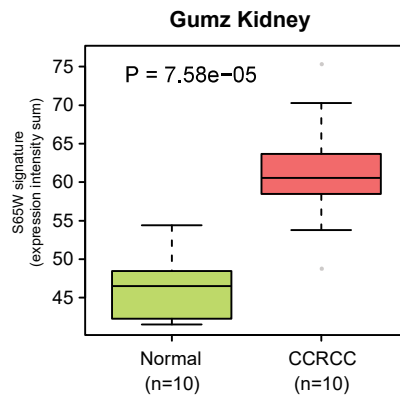

**C**

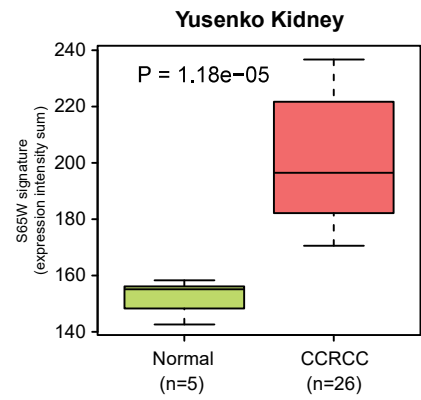

**D**

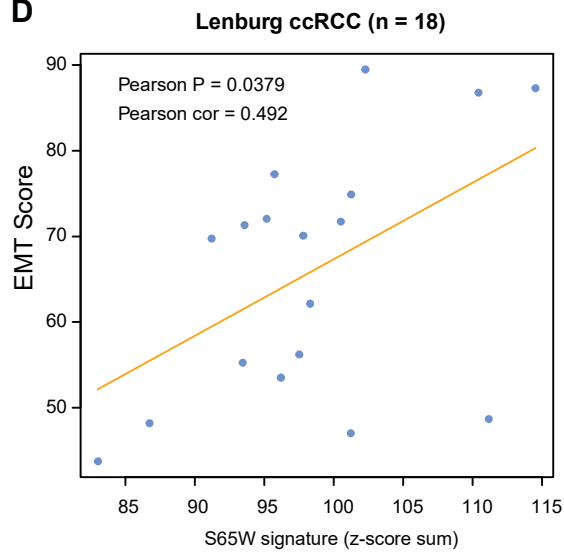

**E**

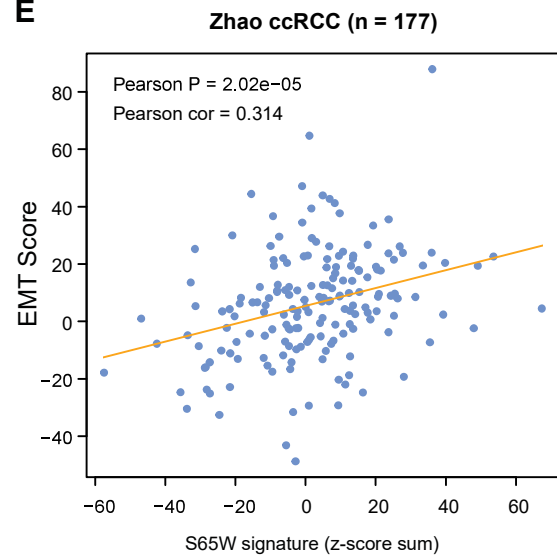

Supplement: Supplementary file 4 — Additional file 4: Figure S4. (A-C) The sum expression levels of VHL-S65W signature gene set are significantly upregulated in human renal cancers compared to normal kidney samples. (D,E) The sum expression levels of VHL-S65W genetic signature positively correlate with EMT scores in the two independent clinical cohorts of ccRCC patient group. [file 13578_2022_790_MOESM4_ESM.pdf]

# Additional file 5

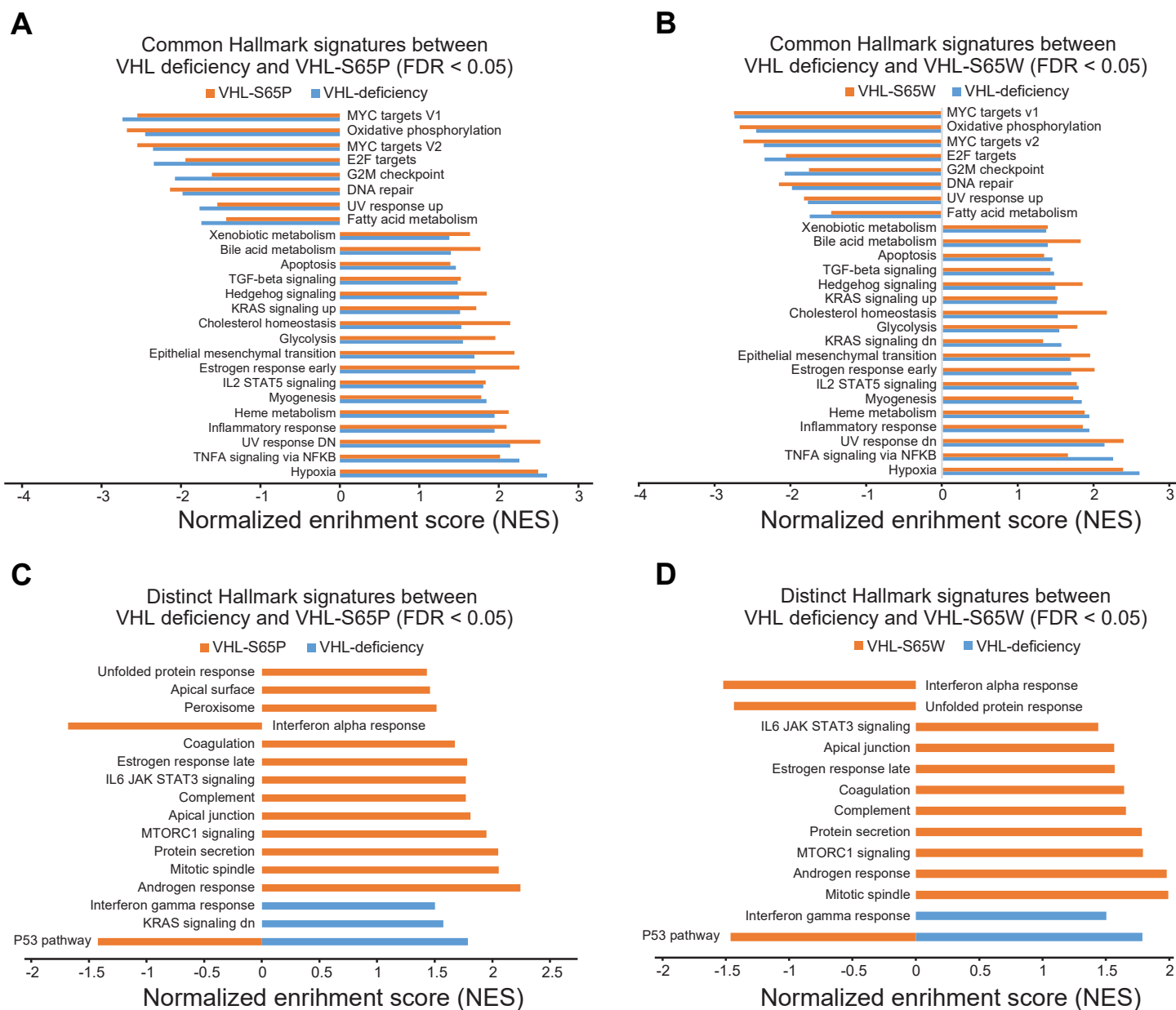

Supplement: Supplementary file 5 — Additional file 5: Figure S5. Gene set enrichment analysis (GSEA) analysis identifies significant common (A and B) and distinct Hallmark signatures between VHL-deficiency and VHL-S65P/W (C and D). Gene signatures with FDR <0.05 are considered as significant [file 13578_2022_790_MOESM5_ESM.pdf]
