# Supplementary material for: VHL Ser65 mutations enhance HIF2α signaling and promote epithelial-mesenchymal transition of renal cancer cells
Source: Cell Biosci. 2022 May 3;12:52. doi: 10.1186/s13578-022-00790-x (PMC9066845; doi:10.1186/s13578-022-00790-x)
Supplement: Supplementary file 6 — Additional file 6: Table S1. The statistical results of VHL mutations S65P and S65W patients. [file 13578_2022_790_MOESM6_ESM.docx]

**Table S1. The statistical results of VHL mutations S65P and S65W patients.**

|  | Total patients | Num. of RCC | Penetrance of RCC | Average onset age of RCC (year) |
| --- | --- | --- | --- | --- |
| S65P | 16 | 7 | 43.57% | 36 |
| S65W | 7 | 4 | 57.10% | 39 |
